# Supplementary material for: Antifungal Activity and Biocontrol Potential of Simplicillium lamellicola JC-1 against Multiple Fungal Pathogens of Oilseed Rape
Source: J Fungi (Basel). 2022 Dec 30;9(1):57. doi: 10.3390/jof9010057 (PMC9860836; doi:10.3390/jof9010057)
Supplement: Supplementary file 1 [file jof-09-00057-s001.zip › jof-2118370-supplementary.pdf]

SUPPLEMENTARY MATERIALS

Antifungal Activity and Biocontrol Potential of *Simplicillium lamellicola* JC-1 against Multiple Fungal Diseases of Oilseed Rape

Wenting Li, Tao Luo, Juncheng Li, Jing Zhang, Mingde Wu, Long Yang and Guoqing Li*

State Key Laboratory of Agricultural Microbiology and Key Laboratory of Plant Pathology of Hubei Province, *Huazhong Agricultural University*, *Wuhan 430070*, *China*

*Corresponding author. guoqingli@mail.hzau.edu.cn

SUPPLEMENTARY FIGURES

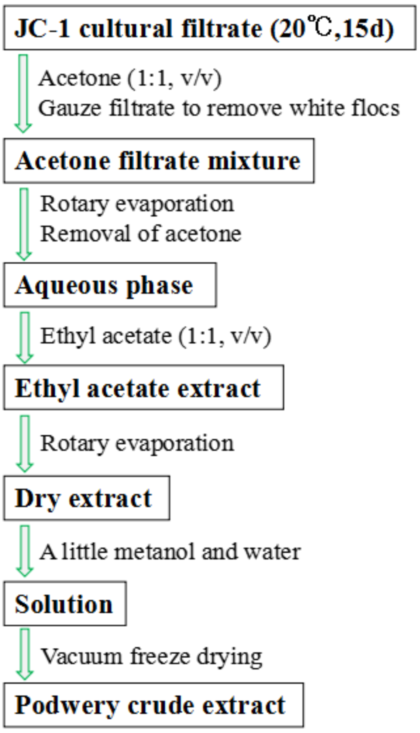

Figure S1. The procedure for obtaining the crude extract from the cultures of *Simplicillium lamellicola* JC-1 in potato dextrose broth.

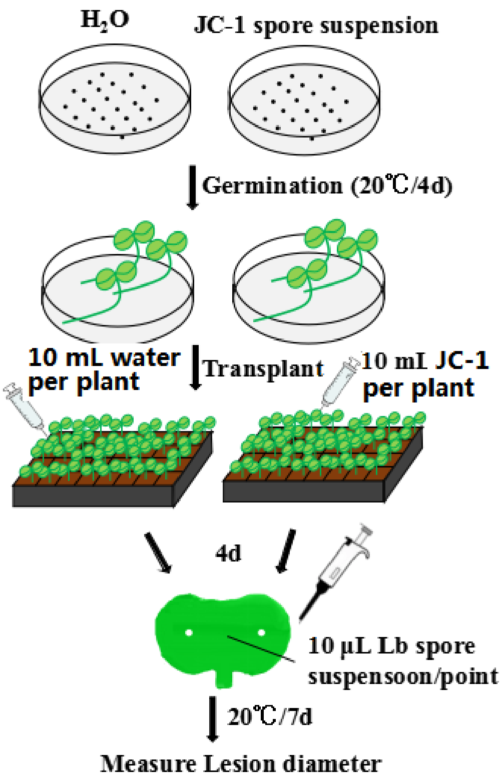

Figure S2. The procedure for determination of the induced systemic resistance of *S. lamellicola* JC-1 against infection by *L. biglobosa* in oilseed rape.

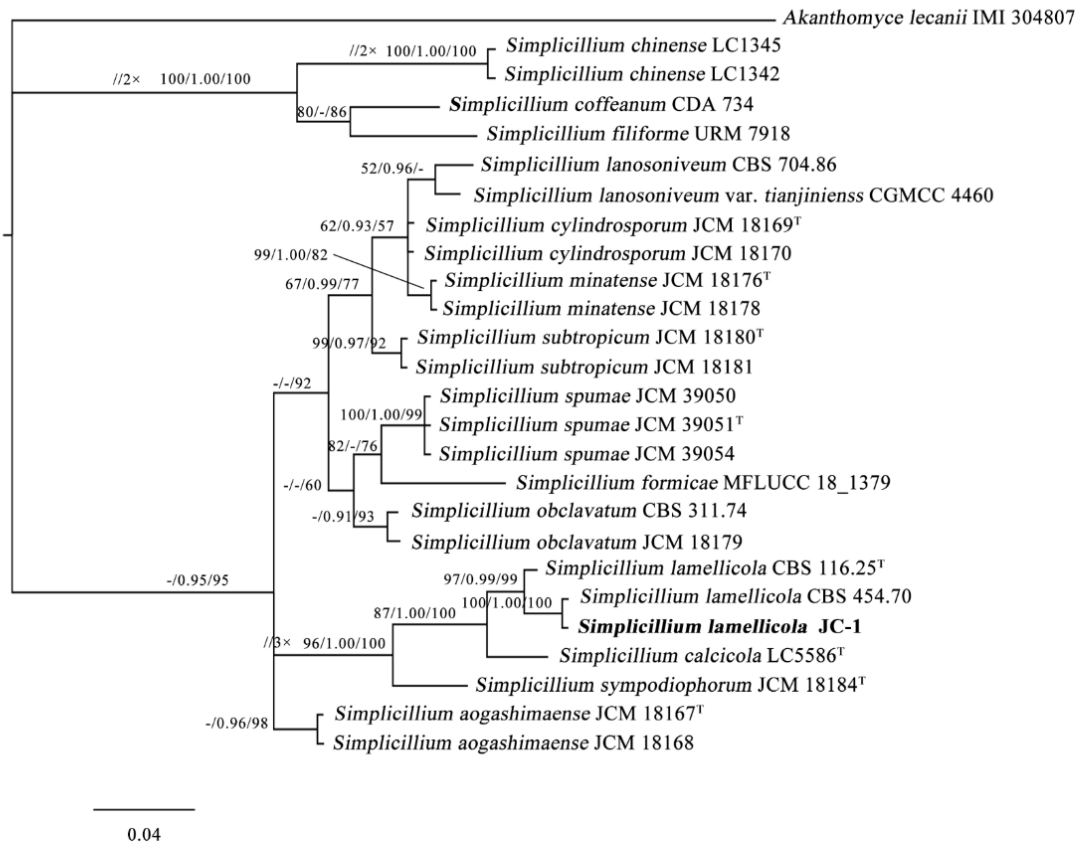

Figure S3. Phylogenetic tree of 26 fungal taxa in the genus *Simplicillium* and *Akanthomyces lecanii* (out-group). The tree was constructed using concatenated sequences of ITS (see Table 1 for GenBank Acc. Nos.). RAxML bootstrap support values (ML) higher than 50, Bayesian posterior probability (PP) higher than 0.90 and maximum parsimony (MP) bootstrap support values higher than 50 were shown at the nodes (ML/PP/MP). Scale bar indicates 0.4% sequence divergence. Type strains labeled with 'T' at the top-right corner.

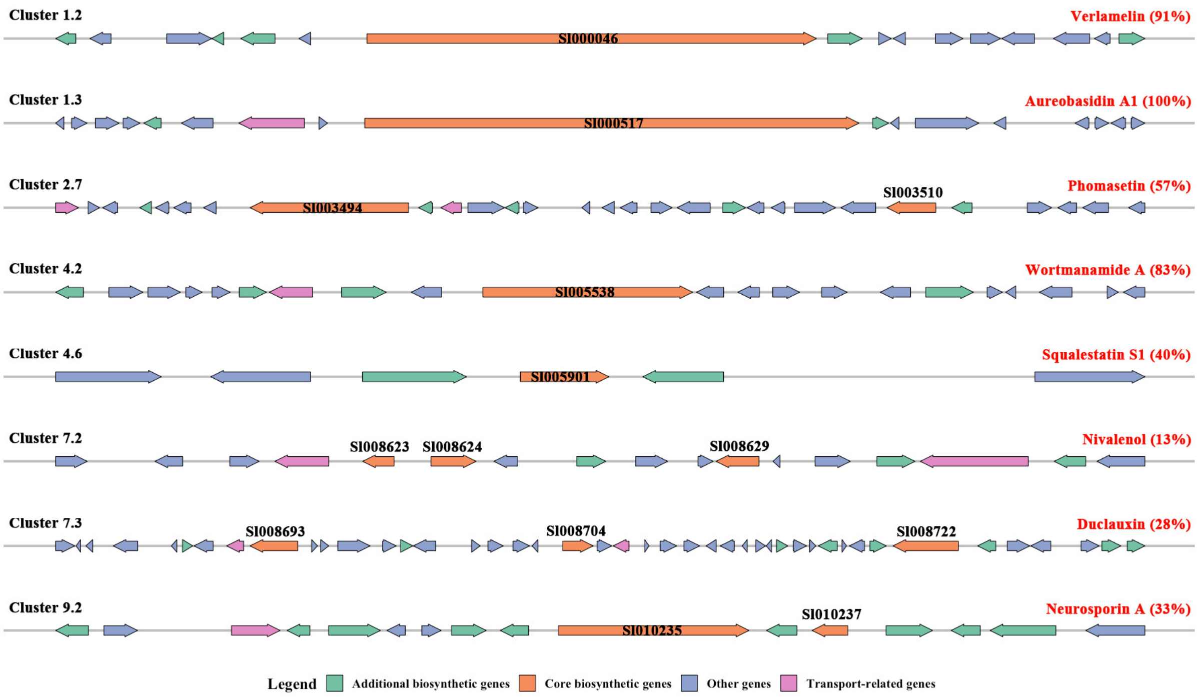

Figure S4. Diagram showing composition of the eight biosynthetic gene clusters for known metabolites in *Simplicillium lamellicola* JC-1 based on antiSMASH.

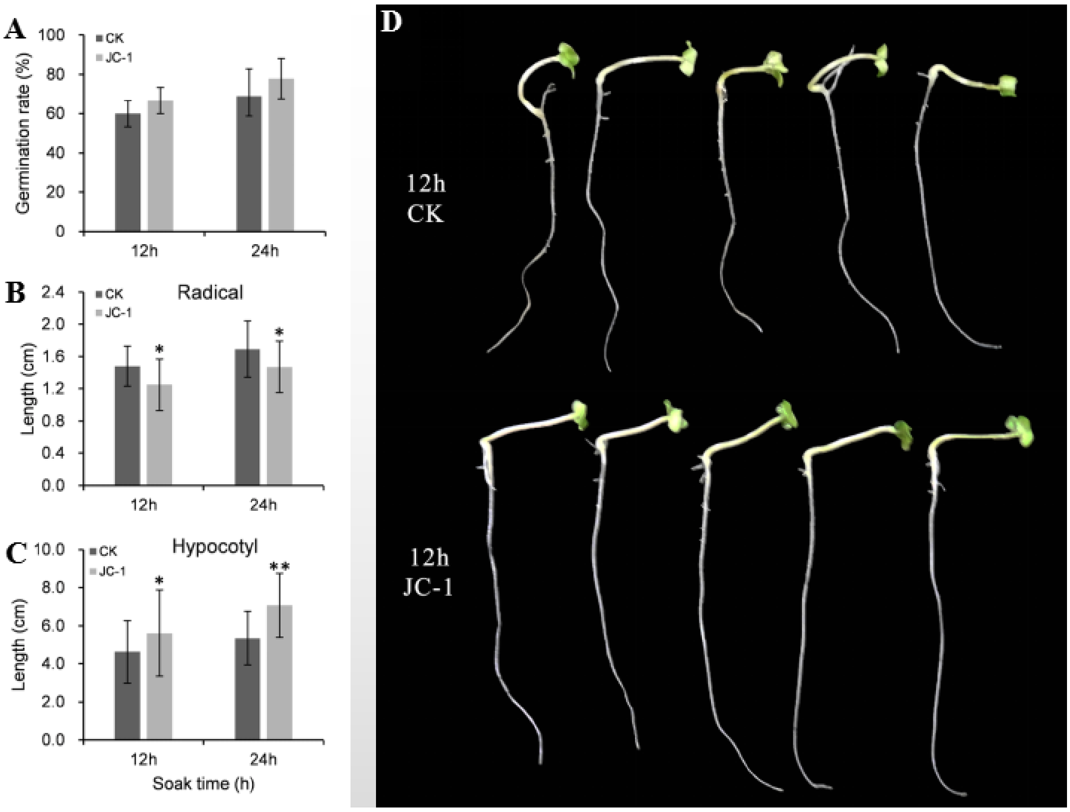

Figure S5. Effect of treatment of seeds of oilseed rape with the conidia of *S. lamellicola* JC-1 on seed germination rate and elongation of the radicles and hypotyls of the resulting seedlings (20°C, 4 d).

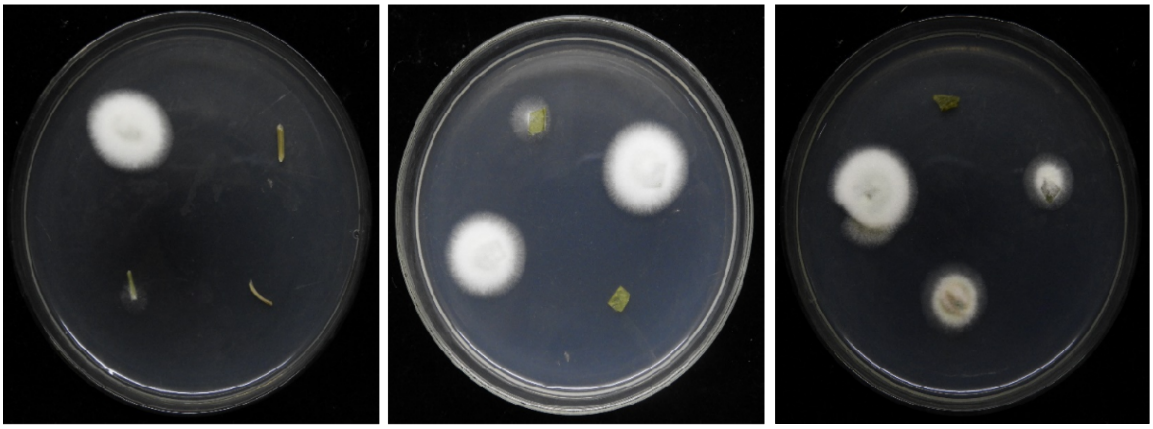

Figure S6. Detection of *S. lamellicola* JC-1 in stems (right dish) and leaves (middle and left dishes) of oilseed rape. Note the whitish colonies of *S. lamellicola*.

SUPPLEMENTARY TABLES

Table S1 PCR primers and their oligonucleotide sequences

Locus[1]	Oligonucleotide Sequence (5' to 3')	Annealing Temperature	Reference[2]
ITS	ITS4: TCCTCCGCTTATTGATATGC<br>ITS5: GGAAGTAAAAGTCGTAACAAGG	49°C	White *et al.*, 1990
SSU	NS1: GTAGTCATATGCTTGTCTC<br>NS4: CTTCCGTCAATTCCTTTAAG	45°C	White *et al.*, 1990
LSU	LR0R: GTACCCGCTGAACTTAAGC<br>LR5: ATCCTGAGGGAAACTTC	49°C	Vilgalys & Hester, 1990
TEF1α	EF1-983F: GCYCCYGGHCAYCGTGAYTTYAT<br>EF1-2218R: ATGACACCRACRGCRACRGTYTG	52°C	Rehner & Buckley, 2005
ACT7	ACT7F: GCTGACCGTATGAGCAAAG<br>ACT7R: AAGATGGATGGACCCGAC	49°C	Peng *et al.*, 2020
CHI	CHI : FGTAGTCATATGCTTGTCTC<br>CHIR: CTTCCGTCAATTCCTTTAAG	47°C	
NCED3	NCED3F: CGATTTGCCTTACCAAGTCAG<br>NCED3R:TTTATCCCTTCCGGTGAGAA	49°C	
PR-1	PR-1F: CATCCCTCGAAAGCTCAAGAC<br>PR-1R: CCACTGCACGGGACCTAC	53°C	

[1]**ITS** = Internal Transcribed Spacer (ITS1-5.8S rDNA-ITS2), **SSU** = Small Subunit ribosomal RNA gene, **LSU** = Large Subunit ribosomal RNA gene, ***TEF1α*** = translation elongation factor 1*α*, ***ACT7*** codes for actin, ***CHI*** codes for chalcone isomerase, ***NCED3*** codes for 9-cis-epoxycarotenoid dioxygenase, ***PR-1*** codes for pathogenesis-related protein 1.

[2]Reference: (1) Peng G *et al. Can J Plant Pathol* 2020, 42: 480–492; (2) Rehner SA, and Buckley E. *Mycologia*, 2005, 97: 84–98; (3) Vilgalys R and Hester M. *J. Bacteriol.* 1990, 172: 4238–4246; (4) White TJ *et al. In*: PCR Protocols: A Guide to Methods and Applications (Innis MA *et al.* eds) Academic Press, San Diego, CA, USA, 1990, pp315–322.

Table S2. Reagents for PCR amplification of ITS, LSU, SSU and *TEF1a*

Reagent	Concentration	Dosage
Forward primer	20 μmol/L	1.0 μL
Reverse primer	20 μmol/L	1.0 μL
DNA template	50 ng/μL	1.0 μL
Master Mix	-	12.5 μL
ddH_2O	-	9.5 μL
Total volume	-	25.0 μL

Table S3. Thermal program for PCR amplification of ITS, SSU, LSU and *TEF1a*

Stage	Temperature	Time	Cycle
Pre-denaturation	95°C	3 min	1 cycle
Denaturation	95°C	30 s	
Annealing	-	30 s	32 cycles
Extension	72°C	1 min	
Final extension	72°C	10 min	1 cycle
Temperature dropping	16°C	5 min	Not applicable

Table S4. Reaction system for DNA elimination in the RNA extracts.

Reagent	Dosage	Temperature	Time
RNA	2 μg		
10×DNaseIBuffer	2 μL	37°C	30 min
Recombinant DNaseI	1 μL		
Stop solution	1 μL	65°C	10 min

Table S5. Reagents for RT-PCR to detect expression of defense-related genes.

Reagent	Concentration	Dosage
Forward primer	20 μmol/L	1.0 μL
Reverse primer	20 μmol/L	1.0 μL
cDNA template	50 ng/μL	1.0 μL
Master Mix	-	12.5 μL
ddH_2O	-	9.5 μL
Total volume	-	25.0 μL

Table S6. Thermal program for quantitative RT-PCR.

Temperature	Time	Cycle
95°C	3 min	1
95°C	10 s	
60°C	30 s	39
72°C	30 s	
95°C	1 min	1
55°C	1 min	1
Melt Curve 55°C to 95°C, increment with 0.5°C	10 s	1

Table S7. Genes clusters for biosynthesis of secondary metabolites (SMs) in *Simplicillium lamellicola* strain JC-1.

Cluster	Type	Position (nt)	Backbone Gene	Putative Protein	Secondary Metabolite	MIBiG Acc. No.	Similarity
1.1	T1PKS NRPS	33680-82407	Sl000016	Putative lovastatin nonaketide synthase	Unknown	–[c]	–
1.2	NRPS	131433-196258	Sl000046	Non-ribosomal peptide synthetase	Verlamelin	BGC0000456	100%
1.3	NRPS	1433867-1503267	Sl000517	Aureobasidin A1 biosynthesis complex	Aureobasidin A1	BGC0000307	100%
1.4	NRPS-like	1970795-2013061	Sl000693	NRPS-like enzyme	Unknown	–	–
1.5	Terpene	2109867-2131080	Sl000745	Isoprenoid synthase	Unknown	–	–
1.6	NRPS	3773786-3821937	Sl001334	Non-ribosomal peptide synthase	Unknown	–	–
1.7	NRPS T1PKS	3935356-3987606	Sl001394	Putative hybrid NRPS/PKS enzyme	Unknown	–	–
2.1	Betalactone	1033569-1058485	Sl002201 Sl002202	Hydroxymethylglutaryl-lyase, Acetoacetyl-synthase	Unknown	–	–
2.2	NRPS	1435270-1534754	Sl002347 Sl002349	Non-robosomal peptide synthetase, Nonribosomal peptide synthase	Unknown	–	–
2.3	NRPS-like	2397011-2439229	Sl002661	Oxygen-dependent choline dehydrogenase	Unknown	–	–
2.4	T1PKS	2680421-2725247	Sl002765	Putative polyketide synthase	Unknown	–	–
2.5	NRPS-like	3093698-3134818	Sl002932	Putative peroxisomal-coenzyme A	Unknown	–	–

				synthetase-like			
2.6	NRPS	3816434-3867433	Sl003210	Nonribosomal peptide synthase	Unknown	–	–
2.7	NRPS T1PKS NRPS-like	4644938-4738920	Sl003494 Sl003510	Putative equisetin synthetase, Putative Linear gramicidin synthase subunit D	Phomasetin	BGC0001738	57%
2.8	T1PKS	4820789-4868697	Sl003552	Polyketide synthase	Unknown	–	–
3.1	T1PKS	1641267-1679314	Sl004278	Methylphloroacetophenone synthase	Unknown	–	–
3.2	Terpene	1,740,519-1,761,688	Sl004310	Geranylgeranyl pyrophosphate synthase	Unknown	–	–
3.3	NRPS NRPS-like	3,601,494-3,656,816	Sl004994, Sl004998	Non-ribosomal peptide synthetase, Nrps-like enzyme	Unknown	–	–
3.4	T1PKS	3674131-3722214	Sl005026	Polyketide synthase	Unknown	–	–
4.1	Terpene	642917-661016	Sl005401	Squalene cyclase	Unknown	–	–
4.2	T1PKS	969343-1014595	Sl005538	Polyketide synthase	Wortmanamide A	BGC0001954	83%
4.3	T1PKS	1184816-1229761	Sl005624	Polyketide synthase	Unknown	–	–
4.4	T1PKS	1365775-1403953	Sl005676	Polyketide synthase	Unknown	–	–
4.5	NRPS T1PKS	1505312-1565249	Sl005729 Sl005730	Nonribosomal peptide synthetase-like Lovastatin nonaketide synthase-like	Unknown	–	–
4.6	Terpene	2065671-2087169	Sl005901	Squalene synthetase	Squalestatin S1	BGC0001839	40%
4.7	NRPS	2482414-2543614	Sl006048	Non-ribosomal peptide synthetase	Unknown	–	–
5.1	NRPS	635243-679402	Sl006597	Nonribosomal peptide synthase	Unknown	–	–
5.2	NRPS T1PKS	2612716-2664965	Sl007294	Polyketide synthase	Unknown	–	–
7.1	NAPAA T1PKS	18904-94369	Sl008578 Sl008589	Non-ribosomal peptide synthetase, Putative acyl carrier	Unknown	–	–
7.2	Fungal-RiPP Terpene	128776-171501	Sl008623 Sl008624 Sl008629	Trichodiene synthase, Alpha-1,3-mannosyltransferase Putative Gamma-glutamyltranspeptidase	Nivalenol	BGC0001277	13%
7.3	NRPS-like T1PKS	303687-415883	Sl008693 Sl008704 Sl008722	Transferase family, Acetyl-CoA synthetase-like, Polyketide synthase	Duclauxin	BGC0001578	28%
8.1	T1PKS NRPS-like, Indole	330549-393056	Sl009359 Sl009365 Sl009366	Putative polyketide synthase, Aromatic prenyltransferase, Acetyl-CoA synthetase-like	Unknown	–	–
8.2	T1PKS	766436-814788	Sl009509	Polyketide synthase	Unknown	–	–
8.3	NRPS-like	1725699-1771470	Sl009893	Aminoadipate reductase enzyme	Unknown	–	–

9.1	NRPS-like	59244-102327	Sl009957	Acetyl-CoA synthetase-like protein	Unknown	–	–
9.2	T1PKS Indole	738964-786458	Sl010235 Sl010237	Polyketide synthase, Prenyltransferase PT1	Neurosporin A	BGC0002729	33%
10.1	NRPS-like	381851-425168	Sl010404	Nonribosomal peptide synthetase	Unknown	–	–

[a]The codes for the gene clusters were named after their positions in the contigs for the genome of JC-1;
[b]T1PKS, Type I polyketide synthase; NRPS, Non-ribosomal peptide synthetase; Terpene, Terpene cluster; Betalactone, beta-lactone containing protease inhibitor; NAPAA, non-alpha poly-amino acids like e-Polylysin; Fungal-RiPP, Fungal RiPP with POP or UstH peptidase types and a modification; Indole, Indole cluster.
[c]–, Not applicable.

Table S8. Putative genes of the verlamelin biosynthesis gene cluster in *S. lamellicola* JC-1.

Gene in JC-1	Predicted function	Homolog in *Lecanicillium* HF627 (GenBank Acc. No.)	Identity	
			nt	aa
Sl000046	Non-ribosomal peptide synthase	*vlmS* (AB862312)	90%	92%
Sl000045	Thioesterase	*vlmB* (AB862314)	92%	96%
Sl000044	AMP-dependent ligase	*vlmC* (AB862315)	90%	96%
Sl005221	Fatty acid hydroxylase	*vlmA* (AB862313)	51%	47%

Note:Three genes (Sl000044, Sl000045 and Sl000046) are located in Contig No. 01, whereas the gene Sl005221 is located in Contig No. 4.
